# Effortful Control and Prefrontal Cortex Functioning in Children with Autism Spectrum Disorder: An fNIRS Study

**DOI:** 10.3390/brainsci10110880

**Published:** 2020-11-20

**Authors:** Karthikeyan Krishnamurthy, Michael K. Yeung, Agnes S. Chan, Yvonne M. Y. Han

**Affiliations:** 1 Department of Rehabilitation Sciences, The Hong Kong Polytechnic University, Hong Kong, China; karthikeyan.krishnamurthy@connect.polyu.hk (K.K.); kin-chung-michael.yeung@polyu.edu.hk (M.K.Y.); 2 Department of Psychology, The Chinese University of Hong Kong, Hong Kong, China; aschan@psy.cuhk.edu.hk

**Keywords:** effortful control, executive function, autism spectrum disorder, connectivity, fNIRS

## Abstract

Effortful control (EC) is an important dimension of temperament, but is impaired in autism spectrum disorder (ASD). While EC is associated with the prefrontal cortex (PFC) functioning in typically developing (TD) children, it is unclear whether EC deficits are associated with PFC dysfunction in ASD. This study examines the relationship between EC and PFC activation and connectivity in children with high-functioning ASD. Thirty-nine right-handed children (ASD: *n* = 20; TD: *n* = 19) aged 8–12 years were recruited. The EC level was assessed with the Early Adolescent Temperament Questionnaire—Revised (EATQ-R), and PFC functioning, in terms of activation and connectivity during a frontal-sensitive (*n*-back) task, was assessed using functional near-infrared spectroscopy (fNIRS). Children with ASD showed a significant deficit in EC and its related constructs (i.e., executive, and socioemotional functions) compared to TD controls. They also showed significantly increased overall PFC activation and reduced right frontal connectivity during the *n*-back task. Among children with ASD, the EC level correlated significantly with neither PFC activation nor connectivity; it significantly correlated with social functioning only. This study demonstrated EC deficits and altered PFC functioning in children with ASD, but the exact neural basis of EC deficits remains to be determined.

## 1. Introduction

Autism spectrum disorder (ASD) is a neurodevelopmental disorder characterized by socio-communicative dysfunction with the presence of repetitive or stereotypic behavioral patterns and interests [1]. These features are escalated in individuals with ASD, as a result of impaired temperamental effortful control (EC) [2,3,4]. EC is defined as “the efficiency of executive attention including the ability to inhibit a dominant response and/or to activate a subdominant response, to plan, and to detect errors” [5]. As human behavior comprises habitual or spontaneous actions, EC is mandatory to inhibit a dominant response and initiate a subdominant response [6]. Therefore, EC becomes a major component in controlling cognitive processes with their associated behavior [5], and serves as a defensive mechanism against compulsive thoughts, while regulating overarching emotions [7].

EC encompasses executive attention, flexibility, and inhibitory control components, which help activate, modulate, or withdraw tendencies pertinent to chosen behavior [5,8,9]. In contrast to EC, executive function (EF) consists of a set of higher-order cognitive processes, including updating (or working memory), inhibitory control, and set-shifting, which support goal-oriented actions [10]. Although EC and EF components conceptually overlap with each other in the self-regulation construct, EC differs from EF primarily in five key areas, including engagement in emotion-dependent contexts, working memory involvement, developmental patterns, adaptive function relationships, and underlying neural substrates [11].

The prefrontal cortex (PFC) has long been implicated in the top-down control of behavior [12]. In healthy children, tasks that engage the core EC components, such as flexibility and inhibitory control, typically activate parts of the PFC, anterior cingulate cortex, and parietal regions [12,13,14]. Additionally, the PFC synchronizes with neighboring regions, while regulating behavior associated with attention and inhibitory control in healthy individuals [15,16]. In the context of EC, one functional near-infrared spectroscopy (fNIRS) study found that parent-report temperamental EC was associated with better performance on a child version of the Stroop task, and with less activation in the dorsolateral PFC during task performance in healthy young children [14]. In another fNIRS study, Fekete and Beacher [17] found that a lower level of EC reported by parents was associated with a decrease in frontal network segregation during movie viewing. Altogether, the literature suggests a link between PFC functioning and EC in healthy children.

Extensive structural and functional imaging studies have implicated abnormalities in the brain, especially the PFC, in ASD [18,19,20]. Functional magnetic resonance imaging (fMRI) studies have found that, compared to typically developing (TD) individuals, individuals with ASD demonstrated altered activation in the PFC and other parietal regions during a variety of EF tasks [20,21]. These individuals also displayed altered connectivity within the frontal lobe and between the PFC and parietal regions. For example, some studies have found that individuals with ASD showed weaker functional synchronization between the cingulo-insular regions and the right lateral frontal and inferior parietal areas [22], between the frontal eye field and intraparietal sulcus [23], and between the right anterior PFC and left visual cortex [24] during inhibitory control and attention-orienting tasks. Notwithstanding the evidence that ASD can be conceived as a disorder of frontal lobe function or connection [18,25], the relationship between PFC functioning and EC in children with ASD is still not clear.

Convergent evidence from human lesion, fMRI, and fNIRS studies have shown that the *n*-back task, which requires participants to judge whether the stimulus they are currently seeing is identical to that presented *n* trials prior, relies critically on the PFC [26,27,28]. Although the *n*-back task is often considered a task for working memory, other cognitive processes, including executive attention and inhibitory control, are subsumed while responding to relevant stimuli and ignoring irrelevant stimuli, respectively [29,30]. The *n*-back task has been utilized to study a wide range of populations, including ASD [31]. Whereas the literature has reported inconsistent task performance results in individuals with ASD [32,33], almost all fMRI and fNIRS studies have found altered patterns of PFC activation and/or connectivity during the *n*-back task in adolescents and adults with ASD [34,35], suggesting that the *n*-back task is sensitive in revealing altered PFC functioning in ASD. Thus, we used the *n*-back task as a probe for PFC functioning in this study to clarify whether impaired EC is related to altered PFC functioning in children with ASD.

As an optical neuroimaging tool, fNIRS uses lights in the near-infrared spectrum (700–1000 nm) to measure changes in the concentration of oxygenated (HbO) and deoxygenated (HbR) hemoglobin that take place at the cortical surface [36,37]. This method has been validated against fMRI [38]. Over the past 10 years, this technique has been widely utilized in ASD research and has shown promise in understanding ASD [39]. As fNIRS is a relatively non-demanding neuroimaging modality for children, we used it to measure PFC activation and connectivity in this study. We hypothesized that, compared to TD children, children with ASD would demonstrate deficits in EC and its related functions (i.e., EF and socioemotional function). We predicted that these children would also exhibit altered PFC activation and connectivity during the *n*-back task (i.e., a frontal-sensitive task). Furthermore, we expected EC deficits to be associated with altered patterns of PFC activation and connectivity during the *n*-back task in children with ASD.

## 2. Materials and Methods

### 2.1. Participants

Participants were recruited from primary schools through an advertisement placed on campus, social media, and sent to schools by post. Consequently, 39 right-handed Chinese children, aged between 8 and 12 years, were recruited, with written informed consent obtained from children and their parents. Twenty children diagnosed with ASD by a psychiatrist and clinical psychologist using the Diagnostic and Statistical Manual of Mental Disorders—5th Edition (DSM-5; [1]) were included in the ASD group. Children with ASD receiving medications were excluded. In addition, 19 age-, handedness-, and IQ-matched children were recruited in the TD group. No children in the TD group had episodes of epilepsy, head trauma, developmental delay, or other neuropsychiatric disorders.

### 2.2. Procedure

The study was conducted in accordance with the ethical principles for medical research involving human subjects declared by Helsinki. The experimental protocol was approved by the Human Subjects Ethics Sub-Committee of the Hong Kong Polytechnic University (ethic approval code: HSEARS20170203004). All children were evaluated independently in two sessions (neuropsychological evaluation, and fNIRS data acquisition), which lasted about 2 h in total, including breaks. Simultaneously, the parents or caregivers of children were interviewed with standardized interviewing protocols, which included the short form of the Early Adolescent Temperament Questionnaire—Revised (EATQ-R), Social Responsiveness Scale—Second Edition (SRS-2), and Autism Diagnostic Interview—Revised (ADI-R). The assessments and interviews were conducted by a clinical psychologist, skilled research assistants, and graduate students.

### 2.3. Measures

#### 2.3.1. Short Form of the Early Adolescent Temperament Questionnaire—Revised (EATQ-R)

The level of EC was measured using the short form of the EATQ-R, which is a standardized parent-rated instrument containing 16 items in three subscales (i.e., activation control, attention, and inhibitory control). The items are rated on a 5-point Likert scale, ranging from 1 (almost always untrue of you) to 5 (almost always true of you) for the direct items, and vice versa for the reversed items. A higher score in each domain indicates a greater ability in EC [40].

#### 2.3.2. d2 Test of Attention

The d2 Test of Attention is a standardized paper-and-pencil test for attention, which involves cancelling out all target letters (i.e., the letter “d” with two dashes positioned above or below) interspersed with nontarget letters (i.e., the letter “d” without two dashes, and the letter “p” with any quantifiable dashes) [41]. The concentration performance index obtained by subtracting the sum of correct responses from the sum of commission errors was adopted as the primary measure [42]. The task takes 4.7 min to complete.

#### 2.3.3. Cambridge Neuropsychological Test Automated Battery (CANTAB)

Three subtests from the Cambridge Neuropsychological Test Automated Battery (CANTAB) were administered via 10.5 inch Apple iPad. The reaction time (RTI) task assesses attention in terms of processing speed (motor and mental) and impulsivity. The task involves holding the response button at the bottom of the screen initially, and, as one of the five circles positioned at the top of the screen flashes yellow, participants are required to tap the highlighted circle (target button) as quickly as possible. The mean reaction times (i.e., mean duration of releasing the response button after stimulus presentation) of five-choice variants were calculated [43].

The multitasking test (MTT) measured selective attention (responds to task-relevant stimuli) and inhibition (ignores task-irrelevant stimuli). In each trial, a leftward- or rightward-facing arrow is either presented on the right or left side of the screen. Meanwhile, a cue is presented at the top of the screen, specifying the arrow’s direction or location. Participants must press the right or left button at the bottom of the screen, in accordance with the arrow’s location or direction, depending on the task cue. The switching block error, denoting the sum of incorrect responses during the block with intermixing task cues, i.e., mean duration of stimuli appearance to pressing button between congruent to incongruent stimuli and vice versa), was adopted as a prime measure in this study. The task included 40 practice and 120 test trials, lasting 8 min [44].

The emotion recognition task (ERT) evaluates the ability to distinguish basic facial expressions. The test requires participants to label photographs of male or female facial expressions presented for 200 ms each, using one of six labels (i.e., sadness, happiness, fear, anger, disgust, and surprise) shown on the screen. There is no time limit for responding. The total number of correct responses was adopted as the primary measure. This task included 5 practice and 90 test trials, lasting 9 min.

#### 2.3.4. *n*-back Task

**Figure 1:**
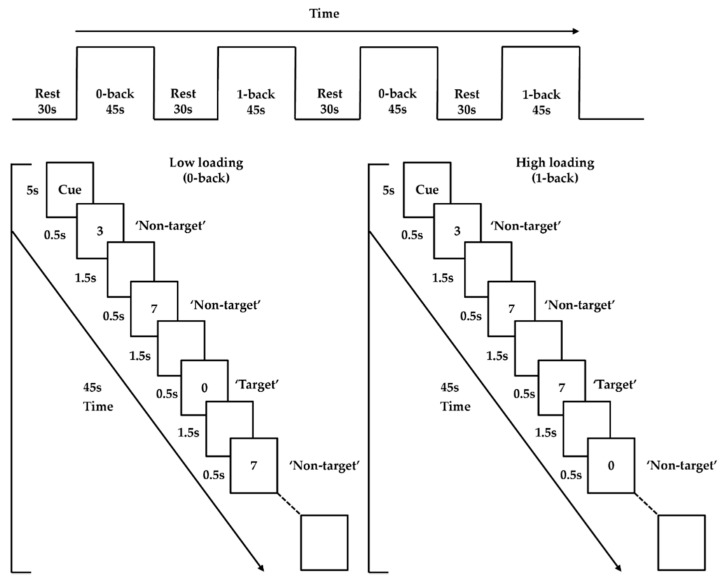
*n*-back task paradigm.

An *n*-back paradigm adopted from previous studies was employed as the activation task to probe PFC functioning [27,45]. It involved two loading conditions (low and high; 0- and 1-back). Trials were presented in 45 s blocks, interleaved with 30 s of rest, for a total duration of 330 s. Each condition was presented twice, and the two conditions were administered in alternating order (i.e., low–high–low–high; or high–low–high–low). The order was counterbalanced across participants to eliminate order effects. Each task block started with 5 s of a visual cue for a condition, followed by 20 (5 target and 15 nontarget) trials presented pseudorandomly. Each trial included a digit that appeared at the center of the screen for 500 ms, followed by an interstimulus interval of 1500 ms (Figure 1). The low-loading (0-back) condition required participants to left-click the mouse with their right index finger when the number “0” (target) was shown, but to right-click the mouse with their right middle finger when other numbers (nontargets) were shown. The high-loading (1-back) condition required participants to left-click the mouse when the number that appeared was the same as the number shown one trial before (i.e., target), but to right-click the mouse for other numbers (i.e., nontargets). E-prime 2.0 software (Psychology Software Tools, Pittsburgh, PA, USA) was utilized to present all stimuli. During task performance, the frontal brain activities were captured using an fNIRS machine.

### 2.4. fNIRS Recording

**Figure 2:**
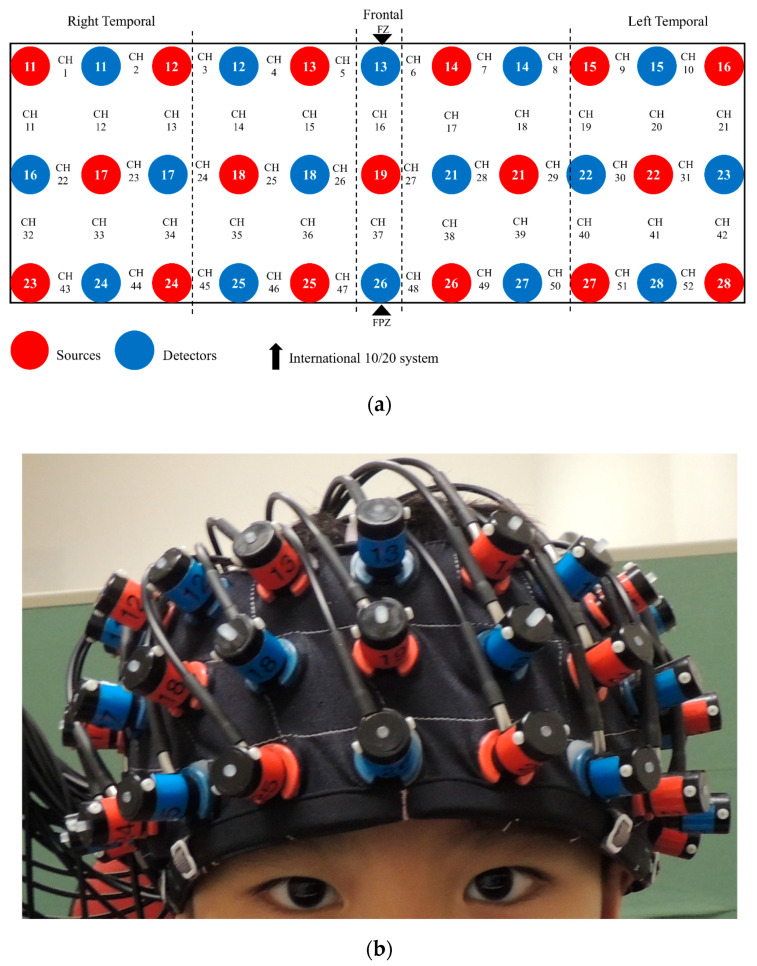
The 3 × 11 montage of the functional near-infrared spectroscopy (fNIRS) system: (**a**) 33 optodes and 52 channels (CH) arrangement; (**b**) placement on head.

During the *n*-back task, fNIRS data acquisition was performed using the Hitachi ETG-4000 machine, which used two wavelengths (695 nm and 830 nm) and sampled data at a rate of 10 Hz. The machine included 33 optodes, including 17 sources and 16 detectors (52 channels), aligned in 3 × 11 montage with a 3 cm source-detector separation (Figure 2). During recording, participants sat on a chair 60 cm away from a 15′’ LCD monitor in a quiet dimly lit room. Participants’ head dimensions (nasion-inion, left-right ear, and head circumference) were measured to facilitate offline spatial registration of NIRS channels [46], in which the channel positions were transformed into the Montreal Neurological Institute (MNI) space, and then projected onto the surface of a volume-rendered children brain template [47,48]. The probe placement regions (forehead) were disinfected with an alcohol pad for better signal quality. A custom-built headband mounted with probes was then placed on the participant’s forehead (covering the PFC). As guided by the standardized reference point on the headband, the center of the bottom optode was anchored at Fpz according to the international 10–20 system. The spatial coordinates of 5 anatomical landmark points (nasion, inion, vertex, and left and right auricular points) and 33 optodes were digitized using a 3D digitizer. Based on the calibration procedure implemented in the acquisition software, good signal quality was ensured before the *n*-back task began.

### 2.5. Data Analysis

Before analyzing the data, normality was checked through Shapiro–Wilk tests. Subsequently, any non-normal data were log-transformed for suitability for parametric testing. If the log-transformed variables still violated the normality assumption, then non-parametric tests were conducted. The data screening and analysis were performed using IBM SPSS Statistics 25 (IBM Corp., Armonk, NY, USA).

#### 2.5.1. Questionnaires and Neuropsychological Measures

The SRS-2 total *T*-score, EATQ-R total score, and other behavioral measures fulfilled the normality assumption. Thus, independent-sample *t*-tests were used for group comparison. However, the behavioral measures of the *n*-back task did not meet the normality assumption even after log transformation. Hence, Mann–Whitney U tests were used to explore the group differences for these variables.

#### 2.5.2. Preprocessing for fNIRS Data

Data preprocessing and analysis were performed using the AnalyzIR Toolbox [49] and Matlab 2019a (The Mathworks, Natick, MA, USA). First, the raw fNIRS data of the *n*-back task were derived in the integral mode after preprocessing with a 0.1 Hz low-pass and a 5 s moving average filter, using the inbuilt Hitachi machine software. The data were then input into the AnalyzIR Toolbox, in which the data were corrected for missing, flat, or saturated channel issues, using default functions. Next, the signals were resampled at 1 Hz, and a 0.1 partial pathlength factor was applied while converting optical density changes into HbO and HbR via the modified Beer–Lambert law [50]. Subsequently, first-level statistical analysis was conducted using the auto-regressive iterative reweighted least-squares (AR-IRLS) approach to estimate activation during task performance [51]. The robust auto-regressive whitened correlation method in the advanced general linear model was also used to estimate connectivity between possible channel pairs. The activation (beta value) and connectivity (*Z*-score) variables were then utilized for group analysis (second-level analysis). We focused on HbO, because, relative to HbR, it has been shown to have a higher signal-to-noise ratio and to correlate more strongly with the Blood Oxygenation Level Dependent (BOLD) signals measured by fMRI [38].

**Figure 3:**
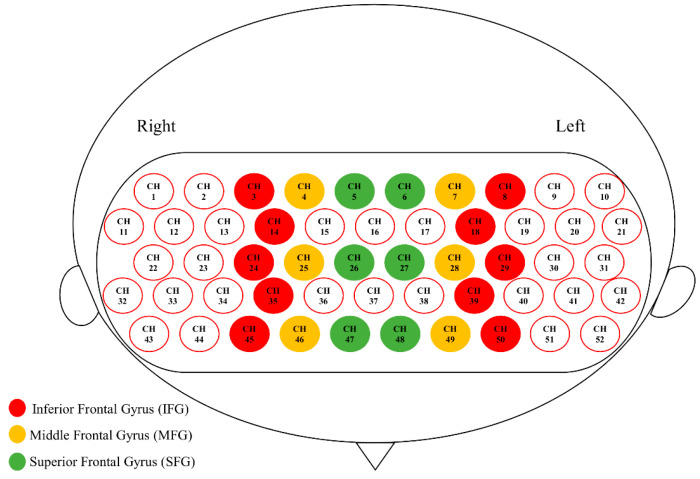
Six anatomically defined ROIs in the prefrontal cortex (PFC).

Spatial registration of channels based on the digitized spatial coordinates (5 reference points and 33 optodes) was performed using Near Infra-red Spectroscopy-Statistical Parameter Mapping (NIRS-SPM; [52]). The output of individual MNI coordinates was further grouped together using BrainNet Viewer [53], and the mean composite estimation was obtained. Using an 80% registration probability, channels that fell in the inferior frontal gyrus (IFG), middle frontal gyrus (MFG), and superior frontal gyrus (SFG) on each side were determined, and the 6 anatomically defined PFC regions were defined as regions of interest (ROI; Figure 3; [54]). Note that some PFC channels were not classified into any ROI, because none of them fell into any ROI with an 80% registration probability, and that temporal lobe channels were not analyzed, because most of them yielded poor signal quality due to poor optode–scalp contact.

#### 2.5.3. fNIRS Data Analysis

For activation, the beta values fulfilled the normality assumption. Hence, a 2 × 2 × 2 mixed multifactorial analysis of variance (ANOVA) was utilized to analyze changes in HbO, in terms of beta values. The statistical model included two within-group factors, loading (low and high) and frontal side (left and right), and one between-group factor (TD and ASD).

**Figure 4:**
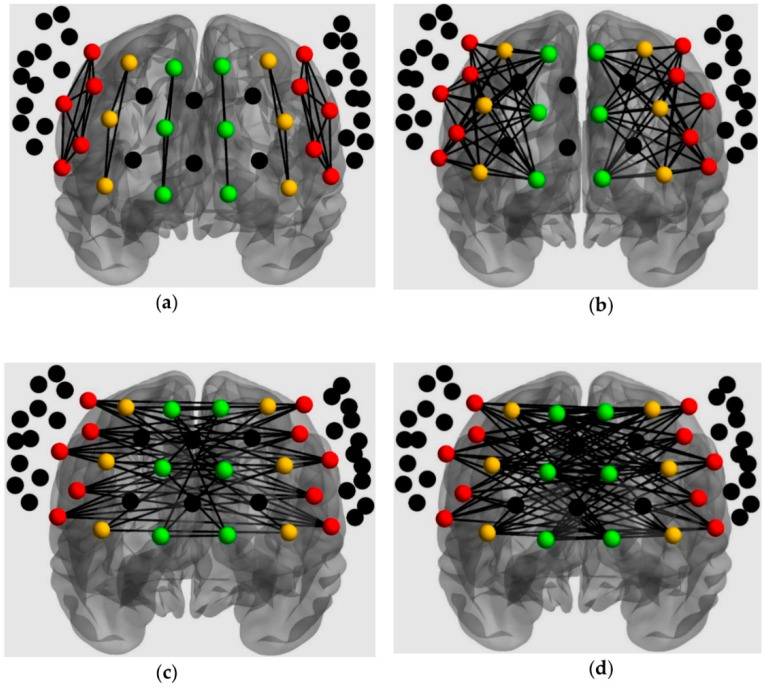
ROIs analysis for connectivity: (**a**) Intrahemispheric connectivity within ROI; (**b**) Intrahemispheric connectivity between ROIs; (**c**) Interhemispheric connectivity within ROIs; (**d**) Interhemispheric connectivity between ROIs.

For connectivity, 6 connectivity patterns were extracted based on the specified ROIs (Figure 4; [54]). Channel pairs were averaged for each connectivity pattern. All connectivity variables (i.e., mean *Z*-scores) fulfilled the normality assumption; hence, intrahemispheric connectivity was analyzed with a 2 × 2 × 2 × 2 mixed ANOVA, which included connectivity pattern (within and between ROI), loading (low and high), and frontal side (left and right) as within-subjects factors, and group (TD and ASD) as the between-subjects factor. Additionally, interhemispheric connectivity was analyzed with a 2 × 2 × 2 mixed ANOVA, which included connectivity pattern (within and between ROI) and loading (low and high) as within-subjects factors, and group (TD and ASD) as the between-subjects factor.

#### 2.5.4. Brain–Behavior Relationship

To explain individual differences in EC among children with ASD, we examined the relationship between measures of EC and measures of PFC functioning and EC-related constructs (i.e., EF and socioemotional measures) for the ASD group specifically. Variables that met and did not meet the normality assumption were analyzed using Pearson’s correlations (*r*) and Spearman’s correlation (*r_s_*), respectively. To reduce the number of comparisons, only variables in which the two groups differed significantly were analyzed.

## 3. Results

### 3.1. Demographic, Intellectual, and Clinical Characteristics

Table 1 shows the demographic, intellectual, and clinical information of the TD and ASD groups. The two groups were matched in age, and IQ, *t*s < 0.42, *p*s > 0.11. Although gender was not matched between groups, *χ*^2^(1) = 6.65, *p* = 0.01, independent-sample *t*-tests revealed no significant differences between male and female TD children in any variable (*p*s > 0.05). As gender was not a confounding factor, it was not controlled for in any subsequent analyses.

### 3.2. EC, Executive, and Socioemotional Measures

The two groups differed significantly in all EC, executive, and socioemotional measures (Table 2), in which the ASD group showed more deficits than TD controls with a large effect size on the EATQ-R total score, *t* (36) = 2.83, *p* = 0.007, the concentration performance index on the d2 Test of Attention, *t* (37) = 2.69, *p* = 0.011, the mean score on the CANTAB reaction time, *t* (36) = 2.67, *p* = 0.011, the switch block error score on the CANTAB multitasking test, *t* (30.37) = 2.46, *p* = 0.019, the total score on the CANTAB emotion recognition task, *t* (35) = 3.48, *p* = 0.001, and the SRS-2 total *T*-score, *t* (35) = 6.22, *p* < 0.001. As the EATQ-R consisted of three discrete constructs, we performed independent-sample *t*-tests to compare the groups on each subscale after adjusting the *p*-value threshold to 0.017. Results showed that the ASD group had more deficits on the attention, *t* (36) = 2.79, *p* = 0.008, and inhibitory control subscales, *t* (36) = 3.05, *p* = 0.005, but not on the activation control subscale, *t* (36) = 1.39, *p* = 0.17, compared to the TD group.

Additionally, children with ASD were slower to respond than TD controls in the low loading condition of the *n*-back task (*U* = 78.00, *p* = 0.001; Table 3), despite comparable accuracy, *p* = 0.79. There was no significant difference in accuracy or mean reaction time between the two groups in the high loading condition, *p*s > 0.074. However, we noted a significant large correlation between accuracy and mean reaction time in the high loading condition among children with ASD (*r* = −0.58, *p* < 0.01), suggesting a speed–accuracy tradeoff. To control for this, the inverse efficiency score (IES) was calculated by dividing the mean reaction time by accuracy for each condition [55,56]. The IES score was also calculated for the low loading condition to facilitate comparison between conditions. The results indicated that the ASD group had significantly poorer performance, in terms of the IES, than the TD group in both loading conditions.

### 3.3. PFC Activation during the N-Back Task

The 2 × 2 × 2 (group × frontal side × condition) mixed ANOVA showed a significant main effect of group, *F*(1,36) = 4.12, *p* = 0.050, *η*_p_^2^ = 0.10, in which the ASD group exhibited more PFC activation (*M* = 0.071, *SE* = 0.018) than the TD group (*M* = 0.019, *SE* = 0.018). No other effects were significant (*p*s > 0.05).

### 3.4. PFC Connectivity during the n-Back Task

#### 3.4.1. Intrahemispheric Connectivity

Results of the 2 × 2 × 2 × 2 (group × connectivity pattern × loading × frontal side) mixed ANOVA conducted for intrahemispheric connectivity (i.e., mean *Z*-scores) are presented in Table 4. The ANOVA demonstrated significant main effects of connectivity pattern, *p* < 0.001, loading, *p* = 0.041, and frontal side, *p* = 0.037. Whereas the main effect of group was not significant, *p* = 0.26, there was a significant interaction between frontal side and group, *p* = 0.005. Independent-sample *t*-tests showed a significant group difference in right intrahemispheric connectivity, *t*(35) = 2.55, *p* = 0.015, in which the TD group exhibited greater right frontal connectivity (*M* = 0.22, *SD* = 0.083) than the ASD group (*M* = 0.15, *SD* = 0.088). There was no significant group difference in left intrahemispheric connectivity, *p* = 0.38.

There was also a significant three-way interaction between connectivity pattern, loading, and group, *p* = 0.024. Follow-up independent-sample *t*-tests, exploring differences between the TD and ASD groups in within- and between-ROI connectivity in the two loading conditions, separately showed a significant difference between the two groups on between-ROI connectivity in the high-loading condition, *t*(35) = 2.17, *p* = 0.037, in which the TD group exhibited greater frontal connectivity (*M* = 0.24, *SD* = 0.037) than the ASD group (*M* = 0.19, *SD* = 0.082). The two groups did not differ significantly in between-ROI connectivity in the low-load condition, or in within-ROI connectivity in either load condition (*p*s > 0.05).

#### 3.4.2. Interhemispheric Connectivity

Results of the 2 × 2 × 2 (groups × connectivity pattern × loading) mixed ANOVA conducted for interhemispheric connectivity (i.e., mean *Z*-scores) are presented in Table 5. The ANOVA demonstrated a significant main effects of connectivity pattern, *p* = 0.003, and loading, *p* = 0.026. The main effect of group was not significant, *p* = 0.15, but there was a significant interaction effect between connectivity pattern and group, *p* = 0.043. Nevertheless, independent-sample *t*-tests revealed no significant difference in either within- or between-ROI connectivity between the two groups (*p*s > 0.05). This interaction effect was driven by the presence of higher between-ROI than within-ROI connectivity in the TD group, but not in the ASD group.

### 3.5. Individual Differences of EC in the ASD Group

We conducted correlation analyses to elucidate the basis of individual differences in EC among children with ASD. These children were found to have poorer performance in all EF and socioemotional measures, increased PFC activation across regions and conditions, and reduced right intrahemispheric connectivity across connection patterns and conditions, compared to TD children. Thus, we examined the correlation between the EATQ-R total score and each EF and socioemotional measure, overall PFC activation, and overall right intrahemispheric connectivity. The EATQ-R total score correlated significantly with the SRS-2 total *T*-score only, *r* = −0.69, *p* = 0.001. No other correlations were significant, *p*s > 0.05.

## 4. Discussion

The primary objective of this study was to elucidate the relationship between EC and PFC functioning in terms of activation and connectivity during a frontal-sensitive (*n*-back) task in children with ASD. We found that the ASD group demonstrated significantly lower levels of EC and its relevant EF and socioemotional measures, compared to TD controls. Children with ASD also exhibited altered PFC functioning, indicated by PFC hyperactivation and reduced right frontal connectivity across *n*-back conditions. We further showed that EC was associated with social skills but not with PFC processing or EF in the ASD group, suggesting that individual differences in EC among children with ASD may be explained by individual differences in social functioning only.

The current findings support our hypotheses. First, the children with ASD presented with more EC deficits than the TD group, such that the EATQ-R differentiated the two groups of children on the attentional and inhibitory control components. The result is consistent with previous findings, in which the parents of adolescents with ASD perceived lower attentional and inhibitory control abilities over a dominating response [3,57,58]. However, in contrast to Samyn and Roeyers’s [57] report of an activation control deficit in ASD, the current finding showed a comparable effect of ASD on activation control, suggesting that the children from the TD and ASD groups had a similar ability to generate and persist with a novel action even when there is an urgency to terminate. The inconsistency may be due to age difference, as they [57] focused on adolescents with ASD, and activation control deficits may become more pronounced with age, due to underdevelopment in ASD.

Our fNIRS findings of reduced right PFC connectivity during the *n*-back task in the ASD group corroborate the “frontal disconnection syndrome” theory of autism, which postulates that frontal disconnection negatively influences the performance of higher-order cognitive tasks [59,60]. Our findings specifically support the underconnectivity theory of ASD, which bridges the neurophysiological basis of complex information processing impairment to its associated frontal lobe dysfunction in individuals with autism [61,62]. The *n*-back task used in our study required participants to monitor and hold onto a piece of information briefly, in accordance with specific loading conditions. It also required participants to respond to interchanging stimuli, involving activating, inhibiting, and switching elements, which necessitate a complex information processing system found to be defective in ASD [63]. As the right lateral frontal lobe has been shown to play an essential role in monitoring [64], hypoconnectivity in the right frontal lobe revealed that children with ASD had difficulty monitoring and processing complex information.

Our findings of a link between EC deficits and social impairment in ASD corroborate the hypothesized role of EC in social affect, empathy, and prosocial behavior, which together assist children in gaining adaptive function [4,65]. However, the present study did not yield significant relationships between EC and task measures of other related constructs (i.e., EF tests, ERT, and *n*-back behavioral measures) or between EC and PFC activation or connectivity. These findings suggest that EC may be a different construct from EF. In addition, questionnaires and behavioral measures may tap distinct response processes. That is, questionnaires include items on real-life behavior that requires individuals to respond using subjective perception or judgement in an open-ended environment and under noncompetitive circumstances, whereas the behavioral measures require individuals to respond on the basis of task performance in a structured setting and under competitive circumstances [66].

Notably, the absence of significant correlations between questionnaires and behavioral measures may be due to the methodological constraints of behavioral measures, which have poor reliability in general [67]. However, the test–retest reliability of the parent-report and behavioral measures used in the present study has been shown to be at least moderate-to-high and comparable with each other (e.g., CANTAB RTI five-choice reaction time in children: *r* = 0.63 [43]; d2 Concentration Performance in adolescents: *r* = 0.74 [68]; EATQ-R score in Chinese adolescents: *r*s from 0.62 to 0.72 [69]; SRS-2 total score in TD and ASD children/adolescents: *r*s from 0.72 to 0.95 [70]. Thus, poor reliability of behavioral measures is not a plausible explanation for the lack of correlations.

Although we found EC deficits and altered PFC functioning in the ASD group, there was a lack of a monolithic relationship between the two, suggesting that individual differences in EC deficits cannot be explained by the degree of overall PFC activation or right frontal underconnectivity among children with ASD. Temperamental EC refers to the ability to inhibit a dominant response to perform a subdominant response in emotionally salient settings [5]. In addition, emotion perception and behavior have been shown to heavily engage the amygdala and anterior cingulate cortex, as well as their interactions with the medial frontal cortex [71,72]. It has long been known that these regions exhibit structural and functional abnormalities in ASD [73]. Thus, it is possible that the EC deficits in ASD are better explained by disturbances in these brain regions and circuits, which remains to be determined.

This study is one of the first to explore EC and its relationship with brain theories and EF in children with ASD. The findings yield valuable evidence that EC deficits and altered PFC functioning are present in these children, but there is no evidence that individual differences in EC can be explained by the extent of altered PFC functioning among children with ASD. In addition, the current study demonstrates temperamental EC deficit and its strong link with social dysfunction in children with ASD, suggesting that EC intervention may be clinically useful to improve real-world social skills in these children. Furthermore, this study generates support for the application of fNIRS to understand ASD [39] and as a cost-effective and user-friendly tool to probe the functional coupling of cortical (but not subcortical) regions during cognitive tasks.

The study has several limitations. First, the small sample size and inclusion of only boys with high-functioning ASD, means that the findings may not be generalized to girls with ASD, low-functioning ASD, or other age groups. Nevertheless, we found that sex was not a confounding factor in any variables among TD children. Second, EC was measured using a parent-report questionnaire alone, and the possibility of parental bias that affects the estimation of the true EC status cannot be ruled out. Third, the *n*-back task had limited difficulty levels, in terms of working memory loading and interference [74], which is necessary to make it understandable to most, if not all, children. Thus, this task may lack optimal sensitivity to assess the level of PFC functioning to be correlated with the EC measure.

## 5. Conclusions

This study showed general deficits in EC and its related constructs (i.e., executive and socioemotional function), as well as altered PFC functioning in children with ASD. It also expands on the previous knowledge of PFC processing during working memory processing among these children and adds converging support for the model of frontal disconnection syndrome and information processing disorder as a neuropathological biomarker of ASD. The relationship between the EC deficit and social dysfunction observed in children with ASD implies that EC may be central to enhancing the social functioning of these children. The lack of a significant monolithic relationship between EC and PFC activation/connectivity among children with ASD warrants further research with the inclusion of a larger sample size and individuals with diverse autistic symptoms, examining the contribution of dysfunction in non-PFC (e.g., limbic) regions or circuits to EC deficits in ASD.

## Figures and Tables

**Table 1:** Demographic, intellectual, and clinical characteristics of the typically developing (TD) and autism spectrum disorder (ASD) groups.

	TD (*n* = 19)	ASD (*n* = 20)	*t*/*χ*^2^/*r*	*p*
	Mean (*SD*)	Mean (*SD*)		
Age (years)	10.28 (0.67)	10.16 (1.04)	0.42	0.68
IQ	108.79 (9.47)	101.65 (16.96)	1.63	0.11
Gender (Males:Females) ^#^	12:07	20:00	6.65	0.010 **
ADI-R Social Interaction ^##^	-	14.20 (7.41)	0.052	0.83
ADI-R Communication ^##^	-	10.75 (5.70)	−0.32	0.18
ADI-R Restricted and Stereotyped Behavior ^##^	-	5.35 (2.70)	−0.30	0.21

Note: *SD*: Standard deviation; ADI-R: Autism Diagnostic Interview—Revised; ^#^ Groups were compared using the chi-squared test with Yates’ correction of the Likelihood Ratio; ^##^ Correlation with Early Adolescent Temperament Questionnaire—Revised (EATQ-R) (total score). ** *p* < 0.01.

**Table 2:** Effortful control, executive and socioemotional functions in the typically developing (TD) and autism spectrum disorder (ASD) groups.

Variables	TD (*n* = 19)	ASD (*n* = 20)	*t*	*p*	*d*
	Mean (*SD*)	Mean (*SD*)			
**EATQ-R ^#^**					
Total	3.18 (0.50)	2.67 (0.61)	2.83	0.007 **	0.91
Attention	3.16 (0.68)	2.54 (0.67)	2.79	0.008 **	0.92
Inhibitory control	3.42 (0.48)	2.79 (0.76)	3.05	0.005 **	0.99
Activation control	2.97 (0.57)	2.68 (0.69)	1.39	0.17	0.46
**D_2_ Test of Attention**					
Concentration performance index	141.2 (20.2)	121.5 (25.0)	2.69	0.011 *	0.86
**CANTAB Reaction Time Task ^#^**					
Mean reaction time (ms)	421.8 (51.2)	468.0 (117.3)	2.67	0.011 *	0.51
**CANTAB Multitasking Test ^#^**					
Switch block error	7.28 (4.39)	12.45 (7.86)	2.46	0.019 *	0.81
**CANTAB Emotion Recognition Task ^#^**					
Total hit rate	23.72 (3.89)	19.00 (4.33)	3.48	0.001 **	1.15
**SRS-2 ^#^**					
Total *T*-score	40.3 (17.4)	87.4 (26.8)	6.22	<0.001 ***	2.09

Note: SRS-2: Social Responsiveness Scale—Second Edition; * *p* < 0.05; ** *p* < 0.01; *** *p* < 0.001; ^#^ Missing data: The EATQ-R was uncompleted for 1 child with ASD; The Cambridge Neuropsychological Test Automated Battery (CANTAB) was not administered to 1 TD child; 1 ASD child did not complete the Emotion Recognition Task; The SRS-2 was uncompleted in 2 TD children.

**Table 3:** *n*-back task performance in the typically developing (TD) and autism spectrum disorder (ASD) groups.

Variables	TD (*n* = 19)	ASD (*n* = 20)	*Z*	*p*	*r*
	Median (95% CI)	Median (95% CI)			
**Mean reaction time (ms)**					
Low load	445.1 (423.8–538.7)	502.1 (517.1–687.1)	3.15	0.001 **	0.50
High load	536.0 (505.0–644.4)	636.3 (603.9–811.6)	1.80	0.074	0.29
**Accuracy**					
Low load	0.97 (0.93–0.97)	0.95 (0.92–0.97)	0.27	0.79	0.043
High load	0.94 (0.87–0.95)	0.89 (0.83–0.93)	0.64	0.53	0.10
**Inverse efficiency score**					
Low load	496.1 (494.7–518.4)	633.8 (621.2–653.9)	5.36	<0.001 ***	0.86
High load	611.3 (608.8–664.8)	794.8 (761.2–875.0)	4.72	<0.001 ***	0.76

Note: ** *p* < 0.01; *** *p* < 0.001.

**Table 4:** Mixed ANOVA (group × loading × frontal side × connectivity pattern) results for intrahemispheric connectivity (i.e., mean Z-scores).

Main/Interaction Effects	Mean (*SE*)	*F*(1,35)	*p*	*η*_p_^2^
Connectivity pattern (within and between ROI)	Within: 0.18 (0.011)	48.28	<0.001 ***	0.58
	Between: 0.22 (0.012)			
Loading (low and high)	Low: 0.21 (0.013)	4.50	0.041 *	0.11
	High: 0.19 (0.010)			
Frontal side (left and right)	Right: 0.18 (0.014)	4.68	0.037 *	0.12
	Left: 0.22 (0.013)			
Group (TD and ASD)	TD: 0.21 (0.015)	1.29	0.26	0.036
	ASD: 0.19 (0.015)			
**Two-way interaction**				
Connectivity pattern *×* loading		3.09	0.088	0.081
Connectivity pattern *×* frontal side		0.037	0.85	0.001
Loading *×* frontal side		2.42	0.13	0.065
Loading *×* group		0.61	0.44	0.017
Connectivity pattern *×* group		0.23	0.63	0.007
Frontal side *×* group		8.98	0.005 **	0.20
**Three-way interaction**				
Connectivity pattern *×* loading *×* frontal side		5.71	0.022 *	0.14
Connectivity pattern *×* loading *×* group		5.61	0.024 *	0.14
Connectivity pattern *×* frontal side *×* group		0.010	0.92	0.000
Loading *×* frontal side *×* group		2.99	0.092	0.079
**Four-way interaction**				
Connectivity pattern *×* frontal side *×* loading *×* group		0.006	0.94	0.000

Note: *SE: Standard error;* * *p* < 0.05; ** *p* < 0.01; *** *p* < 0.001.

**Table 5:** Mixed ANOVA (group × loading × connectivity pattern) results for interhemispheric connectivity (i.e., mean Z-scores).

Main/Interaction Effects	Mean (*SE*)	*F*(1,34)	*p*	*η*_p_^2^
Connectivity pattern (within and between ROI)	Within ROI: 0.19 (0.014)	10.06	0.003 **	0.23
	Between ROI: 0.20 (0.013)			
Loading (low and high)	Low: 0.21 (0.015)	5.43	0.026 *	0.14
	High: 0.18 (0.014)			
Group (TD and ASD)	TD: 0.21 (0.019)	2.14	0.15	0.059
	ASD: 0.17 (0.018)			
**Two-way interaction**				
Connectivity pattern *×* loading		0.75	0.39	0.021
Loading *×* group		1.44	0.24	0.041
Connectivity pattern *×* group		4.41	0.043 *	0.12
**Three-way interaction**				
Connectivity pattern *×* loading *×* group		0.14	0.71	0.004

Note: * *p* < 0.05; ** *p* < 0.01.
